# Pathophysiology and Etiology of Brainstem-Related Dysphagia

**DOI:** 10.3390/audiolres15060153

**Published:** 2025-11-11

**Authors:** Lucia D’Alatri, Maria Raffaella Marchese, Angelo Tizio, Jacopo Galli

**Affiliations:** 1 Unit of Otorhinolaryngology, A. Gemelli University Hospital Foundation IRCCS, 00168 Rome, Italy; lucia.dalatri@unicatt.it (L.D.); jacopo.galli@unicatt.it (J.G.); 2 Department of Head, Neck and Sensory Organs, Catholic University of Sacred Heart, 00168 Rome, Italy; angelo.tizio01@icatt.it

**Keywords:** dysphagia, brainstem, swallowing, central pattern generator, nucleus ambiguus, nucleus tractus solitarius, lateral medullary infarction, amyotrophic lateral sclerosis

## Abstract

Background: Brainstem-related dysphagia represents a complex and severe form of neurogenic dysphagia (ND) arising from lesions that disrupt the central pattern generator (CPG) for swallowing located in the medulla oblongata. Methods: This paper explores the physiological basis of swallowing and its disruption in various brainstem pathologies. Results: The clinical presentation and electrophysiological evaluation of dysphagia are discussed, with a focus on volitional and spontaneous swallowing (SS) and the use of electromyography (EMG)-based assessment techniques. Conclusions: Finally, therapeutic strategies are reviewed, including conventional rehabilitative methods, neuromuscular electrical stimulation, non-invasive brain stimulation, and invasive procedures such as neurobotulinum toxin-A (BoNT-A) injections, balloon dilation, and CP myotomy.

## 1. Introduction

Swallowing is a complex sensorimotor function that requires the coordinated activation and inhibition of over 25 pairs of muscles, along with intact pharyngeal sensation and central neural control originating in the brainstem, the cerebellum, the basal ganglia, and the cerebral cortex. This intricate neuromuscular process involves the engagement of many structures (e.g., mouth, tongue, pharynx, larynx, and esophagus) and it is typically divided into three phases: the oral phase, under voluntary control; the pharyngeal phase, a reflexive process governed by striated muscles; and the esophageal phase, which is involuntary and regulated by the autonomic nervous system.

Neurogenic dysphagia (ND) occurs when neurological damage disrupts any component of the swallowing pathway—from cortical regions through brainstem centers to the peripheral motor units. It is highly prevalent across a range of neurological diseases (e.g., stroke, Parkinson’s disease, dementia, amyotrophic lateral sclerosis—ALS, and neuromuscular disorders), and represents a significant burden not only for patients but also for healthcare professionals and society as a whole.

The consequences of ND can be severe, leading to malnutrition, dehydration, and aspiration pneumonia, which are among the leading causes of morbidity and mortality in patients with chronic and acute neurological illness. In slowly progressive disorders, ND may begin subtly, with a subclinical phase before overt symptoms emerge. This underlines the importance of early diagnosis, which can allow for timely initiation of rehabilitative, medical, or surgical interventions aimed at preventing complications.

The phenotypic expression of ND varies significantly depending on the underlying neurological pathology. For example, the extrapyramidal motor dysfunction in Parkinson’s disease leads to a different dysphagia pattern compared to the pharyngeal hypoesthesia observed in stroke patients. Despite these clinical distinctions, most classification systems focus mainly on the severity of dysphagia—particularly in terms of penetration and aspiration—rather than on its phenomenological features (e.g., timing, coordination, or sensory deficits). This limitation in classification can hinder the development of more personalized therapeutic approaches.

Among the neurogenic causes, lesions of the brainstem are particularly prone to producing severe forms of dysphagia. This is because the central pattern generator (CPG) for swallowing, responsible for generating and coordinating the swallowing sequence, is localized within the brainstem.

In stroke of medulla oblongata, the prevalence of dysphagia can reach up to 80–90% during the acute phase [1] and even in patients who experience recovery, residual deficits may persist. Similarly, lesions in the pons can disrupt the bilateral coordination of the CPG for swallowing, further increasing the risk of aspiration.

Brainstem-related dysphagia is typically more severe and persistent compared to that related to cortical lesions. Indeed, because of the anatomical convergence of both afferent and efferent pathways for swallowing in the brainstem, even small lesions can have disproportionately large clinical effects often requiring instrumental assessment, nutritional support (e.g., artificial feeding via nasogastric tube or percutaneous endoscopic gastrostomy -PEG), and intensive rehabilitation.

This paper aims to illustrate the basic physiology and anatomy of swallowing, the role of electromyography (EMG)-based techniques in the clinical assessment, and the phenotypic differences in ASL and brainstem stroke-related dysphagia. Finally, a brief review of the available therapeutic strategies is reported.

## 2. Basic Physiology and Anatomy Phases of Swallowing and Brainstem Control

Swallowing consists of three main phases: oral, pharyngeal, and esophageal.

The oral phase of swallowing is under voluntary control and involves both the preparation and the propulsion of the bolus into the oropharynx. This phase is primarily regulated by cortical and subcortical centers, which coordinate the complex motor activity needed to manipulate the bolus within the oral cavity and initiate its movement toward the throat.

In contrast, the pharyngeal phase begins reflexively as soon as the bolus enters the oropharynx. It is characterized by rapid and stereotyped motor sequences that are entirely controlled by the brainstem’s central pattern generator (CPG) for swallowing. The primary function of this phase is to ensure the safe and efficient passage of the bolus through the pharynx while simultaneously protecting the airway from aspiration. The pharyngeal phase involves a precisely timed activation of multiple muscles to close the nasopharynx (through soft palate elevation), retract the tongue base, elevate and close the larynx, relax the upper esophageal sphincter (UES), initiate pharyngeal peristalsis. Timing is essential. Improper sequencing or delayed activation can lead to aspiration or bolus retention. The pharyngeal phase is the critical juncture for aspiration prevention and is most commonly affected in ND.

The esophageal phase is entirely involuntary and governed by autonomic processes. During this phase, the bolus is propelled toward the stomach through coordinated peristaltic waves. These wave-like muscle contractions ensure efficient transport of the ingested material through the esophagus. This activity is modulated by both the brainstem and the enteric nervous system, which together regulate the timing and strength of the peristaltic movements to facilitate safe and effective bolus transit into the stomach.

Swallowing is critically dependent on the integration of intact sensory feedback, precise neural control, and dynamic adaptation to internal and external stimuli. At the core of this neuromotor process lies the brainstem, which not only constitutes the principal integrative center responsible for regulating the involuntary components of swallowing—namely, the pharyngeal and esophageal phases—but also is involved in the control of the stereotyped motor activity that characterizes the oral phase. Indeed, although the oral phase is voluntarily initiated, it is nevertheless executed via subcortical mechanisms governed by specialized circuits collectively referred to as the swallowing CPG.

The swallowing CPG constitutes a modular and hierarchically organized system that coordinates sensory input, processes this information through interneuronal networks, and delivers structured motor output to the relevant musculature. This system can be functionally conceptualized as comprising three discrete but interconnected levels:

1. Afferent and Descending Input Level

This level includes peripheral sensory afferents from the oral cavity, the pharynx, and the larynx, as well as descending signals from cortical and subcortical motor centers. Sensory input travels via cranial nerves—primarily the trigeminal (V), facial nerve (VII), glossopharyngeal (IX), and vagus (X)—and terminates in the nucleus of tractus solitarius (NTS), a central hub within the dorsomedial medulla. The NTS acts as the primary integrative center for afferent feedback and descending voluntary control, modulating the initiation and adaptation of the swallowing reflex in response to sensory stimuli such as bolus consistency, volume, and temperature.

2. Organizational or Premotor Level (The Core CPG Network)

Situated within the medulla oblongata, this level includes interconnected interneurons that comprise the functional core of the swallowing CPG. It consists of two major neuronal assemblies: the dorsal swallowing group (DSG), located in and adjacent to the NTS and the dorsal medullary reticular formation, and the ventral swallowing group (VSG), located ventrolaterally above the NA. The DSG contains generator neurons responsible for initiating, timing, and shaping the rhythmic and sequential motor output that characterizes each phase of swallowing. Once the DSG establishes the swallow pattern, it activates the VSG, whose switching neurons distribute this pattern to the appropriate motor nuclei. The DSG and the VSG communicate extensively, with the VSG serving as the interface between premotor control and motor execution through the engagement of cranial (V, VII, IX, X, XII) and spinal nerves (C1–C3).

3. Efferent Output Level

**Figure 1:**
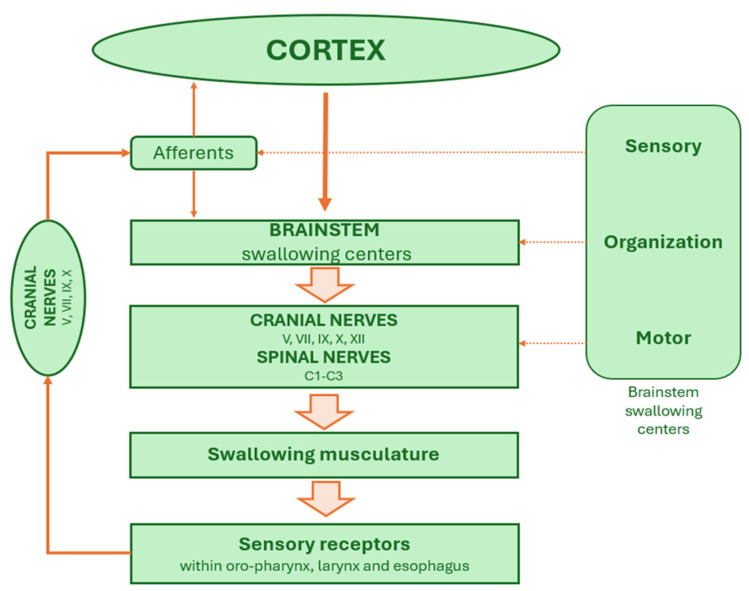
The brainstem swallowing network.

The final level comprises the cranial motoneuron pools located in nuclei of cranial nerves V, VII, IX, X, and XII, which are responsible for innervating muscles of mastication, facial expression, soft palate, pharynx, larynx, and esophagus. These motor neurons execute the highly coordinated muscle contractions required for bolus propulsion and airway protection during swallowing (Figure 1).

Each hemisphere of the medulla oblongata contains a hemi-CPG for swallowing. In normal physiological conditions these bilateral units are synchronized through commissural fibers that ensure rapid and coordinated cross-talk. Sensory information from the oral cavity and pharynx are processed in the NTS and relayed to both the ipsilateral and contralateral CPG networks. The ipsilateral hemi-CPG is primarily responsible for initiating the swallowing response, while synchronization with the contralateral side ensures symmetrical activation of muscles across the midline. This bilateral coordination is essential for the precise control of rostro-caudal and lateral muscle activation during the pharyngeal and esophageal phases. Unilateral lesions of the medulla oblongata, such as those seen in Wallenberg syndrome, can disrupt the function of one hemi-CPG, further highlighting the critical importance of bilateral integration for safe and effective swallowing.

The NA houses the lower motor neurons that innervate the pharyngeal and laryngeal muscles and receives processed sensory inputs from the NTS. Along with the dorsal motor nucleus of the vagus, the NA contributes critically to the execution of motor patterns associated with the pharyngeal and esophageal phases. The integration between the NTS and NA forms the core of the brainstem circuitry required for generating the fixed motor sequence characteristic of the reflexive swallow.

Despite the stereotyped nature of the pharyngeal reflex, the swallowing CPG is highly plastic and adaptive. As emphasized by Lang and colleagues (2009) [2], its motor output can be modulated according to bolus characteristics, such as volume, viscosity, and temperature. Moreover, higher cortical centers—including primary motor cortex, insula, and anterior cingulate gyrus—can influence CPG activity, particularly during voluntary swallows. The adaptability of the system also extends to environmental and postural conditions, highlighting the interplay between reflexive and volitional control mechanisms.

Neurophysiological and electrophysiological studies have demonstrated that the brainstem contains intrinsic timing circuits capable of generating swallowing patterns even in the absence of peripheral sensory inputs. In both human and animal models, rhythmic activity associated with swallowing persists following sensory deafferentation or muscle paralysis, suggesting that premotor interneurons within the medulla oblongata are enough to generate and maintain the motor timing for each of the three swallowing phases [2].

Interestingly, even the oral phase—although consciously initiated—relies on patterned motor output driven by brainstem circuits located in the reticular formation and trigeminal motor nucleus. Observations in anencephalic neonates, who can generate rhythmic sucking and swallowing movements, despite the absence of cortical structures, further support the subcortical origin of these patterns. Thus, the swallowing CPG possesses a remarkable degree of autonomy in regulating essential behaviors [2]. It is also important to note that each phase of swallowing is functionally distinct yet dynamically interlinked through intra-phase and inter-phase reflexes. These include mechanisms such as secondary peristalsis, reflexive swallow initiation, failed-swallow responses, and swallowing inhibition. All these mechanisms contribute to the system’s robustness and flexibility in variable physiological conditions.

Within the NTS, premotor neurons are known to integrate convergent inputs originating from both cortical and peripheral sources that contribute to the initiation of swallowing [3,4]. This integration is particularly critical for the execution of volitionally initiated swallowing. During such feeding behaviors, descending cortical signals are believed to reach the NTS as the first step in triggering the swallow response. Consequently, sequential actions such as eating and drinking can be voluntarily initiated or modulated by the cerebral cortex through its influence on the brainstem’s CPG network [5,6,7,8]. Therefore, in the case of voluntary swallowing, cortical and subcortical regions implicated in deglutition primarily function to initiate the swallowing process and to govern the onset of its motor sequence—particularly the oral phase [6]. Following this initial cortical input, the remainder of the swallowing sequence—namely, the pharyngeal and esophageal phases—is executed in a stereotyped manner, without requiring further cortical involvement [3,5].

Unlike volitional swallowing, which is consciously initiated, spontaneous swallowing (SS) (e.g., the involuntary act of swallowing saliva that occurs without conscious awareness) functions as a fundamental reflex mechanism that is under the control of the medullary components and largely independent of cortical inputs. In addition to the medullary nuclei, the pontine trigeminal nucleus and the surrounding reticular formation are also implicated in the regulation of SS. This suggests a broader pontomedullary network underlying SS control [7,8].

The role of SS is to maintain oropharyngeal hygiene and to protect the upper airways from potential aspiration of saliva or residual food particles [6]. In awake individuals, it typically occurs at a frequency of approximately once per minute [9,10], although reported rates may vary based on individual physiology and methodological differences across studies [11,12]. Factors such as salivary gland activity, which fluctuates in response to oral stimulation, meal ingestion, and hydration, significantly influence the frequency of SS. For instance, salivary swallowing rate increases following food or fluid intake [13], while they decrease significantly during sleep, particularly during slow-wave (non-REM) sleep stages [10,14]. Neurophysiological studies have shown that during sleep SS is often associated with transient EEG arousals, indicating a link between arousal mechanisms and SS [10,14]. The decreased rate of SS during sleep may compromise the upper airways protection. Indeed, it has been reported that silent aspiration of pharyngeal secretions during sleep is a common occurrence in healthy adults, underscoring the physiological vulnerability during this period [15]. Although the precise pacemaking mechanism responsible for SS is embedded within the medullary CPG, yet its alterations may also involve subcortical and extrapyramidal systems [16]. For example, SS rate declines with age, a change that may reflect both peripheral and central nervous system alterations [9]. Recently, SS rate has emerged as a potential non-invasive biomarker for dysphagia screening [17], and an association between its reduced rate and post-stroke oropharyngeal dysphagia has been described [18]. However, despite it shows promise as a clinical tool, standardization of methods and large-scale validation are imperative for its widespread adoption. In the future, the SS rate could complement existing assessments, thereby enabling earlier and more accurate management of oropharyngeal dysphagia [17].

In sum, SS represents a vital, though often overlooked, component of the swallowing repertoire. Governed primarily by subcortical and brainstem mechanisms, it ensures continuous airways protection and fluid clearance without conscious engagement. Given its modulation across circadian rhythms and its sensitivity to physiological and pathological states, SS provides valuable insight into the integrity of the neural circuits that support swallowing, and deserves further investigation, particularly in vulnerable populations such as the elderly or neurologically impaired.

## 3. Electromyographic Evaluation of Brainstem Function

While SS is an important parameter in evaluating ND, volitional swallowing should be the primary focus of neurophysiological assessment. In addition to the classic instrumental evaluation of dysphagia (e.g., flexible endoscopic evaluation of swallowing—FEES; videofluoroscopy), EMG-based techniques may be used for the assessment of ND. To appreciate the role of EMG in oropharyngeal swallowing, a brief review of the sequential events involved in volitional swallowing is necessary.

The swallowing process begins with the initiation of the swallowing reflex in the oropharyngeal cavity, triggered by the presence of a bolus. This sensory input ascends to the brainstem and cerebral cortex. The second phase involves elevation and closure of the larynx and soft palate to protect the airway, along with contraction of the submental and suprahyoid muscles located beneath the chin. The third step is the generation of propulsive tongue force and pharyngeal constrictor activity that propels the bolus through the pharynx. Finally, the cricopharyngeal muscle of the UES—normally closed at rest—relaxes and opens to allow bolus passage into the esophagus.

To evaluate these events and associated swallowing disorders, several EMG-based techniques can be applied in what is termed “single bolus analysis” [16]:

1. Submental/Suprahyoid EMG (SM-EMG): records the onset and duration of pharyngeal swallowing. These muscles fire synchronously at the initiation of oropharyngeal swallowing. Surface electrodes are typically placed 1 cm lateral to the midline on both sides beneath the chin.
2. Laryngeal Movement Monitoring: a piezoelectric sensor positioned between the thyroid and cricoid cartilages tracks vertical laryngeal motion during swallowing. Alternatively, surface EEG electrodes may be placed above the thyroid cartilage to monitor timing of laryngeal elevation during the pharyngeal phase.
3. Cricopharyngeal Sphincter EMG (CP-EMG): EMG activity of the UES can be recorded using a concentric needle electrode inserted percutaneously and directed postero-medially in the neck.
4. Perioral and Masseter EMG for spontaneous swallowing: surface electrodes placed on the lip muscles and masseter are used to assess spontaneous swallowing.

Besides the Single Bolus Analysis, the Dysphagia Limit test is another interesting tool in diagnosing ND [19]. This test evaluates a subject’s maximum capacity for swallowing water in a single attempt. Using SM-EMG and a laryngeal movement sensor—and optionally respiratory signals—this test quantifies swallowing efficiency. Healthy adults can swallow 20 mL or more in a single effort, whereas ND patients often fail to do so. In such cases, smaller volumes (5, 10, 15, and 20 mL) are administered incrementally. Patients who divide even modest volumes into multiple swallows—a phenomenon known as piecemeal deglutition—are considered dysphagic. Any repetition of swallowing at or below 20 mL is indicative of pathology [20,21].

In sum, EMG is a non-invasive method to assess swallowing that allows one to understand the pathogenesis of the disorder and to diagnose and follow-up the patient objectively and quantitatively. Moreover, it has recently been suggested that EMG—based techniques may be useful for ND screening [22] and may also be used as a biofeedback during swallowing exercises [23,24].

## 4. Pathophysiology of Disease Brainstem Lesions and Dysphagia

Dysphagia is a frequent and often severe symptom observed in several brainstem diseases (Table 1).

Its pathogenesis, phenotypic manifestations, and clinical implications may vary greatly depending on the underlying pathology. A nuanced understanding of these differences is essential for accurate diagnosis, targeted intervention, and appropriate management of patients.

The lower brainstem—particularly the medulla oblongata—is a vital structure, and acute lesions in this region are often life-threatening. Consequently, few diseases involving the pontobulbar area allow for prolonged survival or sufficient time for detailed clinical and instrumental evaluation. Among these, two conditions are particularly amenable to diagnosis and patient care: lateral medullary infarction (LMI), also known as Wallenberg syndrome, which generally has a favorable prognosis, and ALS, which unfortunately carries a poor prognosis.

### Clinical and Instrumental Findings

In patients affected by ALS, dysphagia typically presents as a complex swallowing disorder, representing the predominant phenotype in approximately 48% of patients, according to the classification proposed by Warnecke et al. (2021) [25]. This complexity stems from the widespread degeneration of both upper and lower motor neurons, leading to a combined bulbar and pseudobulbar dysfunction. FEES findings in ALS often reveal a constellation of impairments including premature bolus spillage, pharyngeal residue in both the valleculae and piriform sinuses, and reduced pharyngeal contractility. One key characteristic of dysphagia in ALS is that pharyngeal impairment is never isolated, but rather always occurs in conjunction with oral phase dysfunction, reflecting the disease’s diffuse impact on the motor system. Moreover, ALS patients often exhibit disrupted coordination between swallowing and breathing. A significant finding reported by Warnecke et al. (2021) is the presence of arrhythmic or irregular swallowing intervals during spontaneous swallowing in up to 43% of ALS cases [25]. These disruptions are thought to reflect dysfunction within the CPG for swallowing. Although respiratory function itself may remain preserved early in the disease course, the inability to maintain the brief apneic period required during swallowing often leads to poor synchronization between respiratory and swallowing events, thereby increasing the risk of aspiration.

The tongue contributes actively to both the oral and pharyngeal phases of swallowing by shaping, positioning, and propelling the bolus through the oral cavity and into the pharynx. Recently it has been shown that Maximum Tongue Pressure (MTP) is significantly reduced in patients with ALS. This finding is associated not only with disease progression and bulbar involvement, but also with overall prognosis and survival [26]. A reduced MTP has been particularly associated with the presence of post-swallow residue in the pyriform sinuses, indicating impaired swallowing efficacy, especially with more viscous consistencies and larger bolus volumes, which demand greater muscular effort. Interestingly, no clear correlation was found between MTP and residue in the valleculae or with penetration and aspiration events. Another important aspect emerging is tongue endurance, defined as the duration one can sustain submaximal tongue pressure [27]. Tongue endurance was found to be reduced in approximately half of the patients with ALS and was associated with a higher frequency of penetration events. This reduction likely reflects bulbar fatigue, a common and debilitating symptom in ALS, that may worsen during meals, thus exacerbating dysphagia symptoms. These findings carry significant clinical implications. Indeed, incorporating the assessment of tongue pressure and endurance into dysphagia evaluations may offer valuable insights into the patient’s functional reserve and eating-related fatigue.

EMG studies in ALS further support the presence of neurogenic swallowing dysfunction. One notable electrophysiological feature is the hyperreflexic CP muscle, characterized by a shortened EMG pause, premature sphincter closure before the laryngeal descent, and paradoxical EMG bursts during what should be a period of silence. Interestingly, despite these functional disturbances, the CP muscle and its motor units generally remain structurally intact, suggesting that the observed abnormalities may be due to impaired corticobulbar and premotor modulation rather than direct lower motor neuron degeneration of the CP muscle. This phenomenon mirrors findings in other ALS-affected muscles, such as the anal sphincter, which are also typically spared from early lower motor neuron involvement. Consequently, even when swallowing is generated by the medullary CPG, the hyperreflexic CP sphincter may act as a mechanical barrier, impeding effective bolus passage. In advanced ALS, such dysfunction necessitates the use of PEG to ensure adequate nutrition and reduce the risk of aspiration pneumonia.

Brainstem strokes account for about 9 to 21.9% of strokes [28]. As swallowing-related structures are located in the lateral medulla, ND is a common clinical feature of LMI (51% to 100% incidence) [29], with aspiration occurring in about 40% of cases [30].

**Figure 2:**
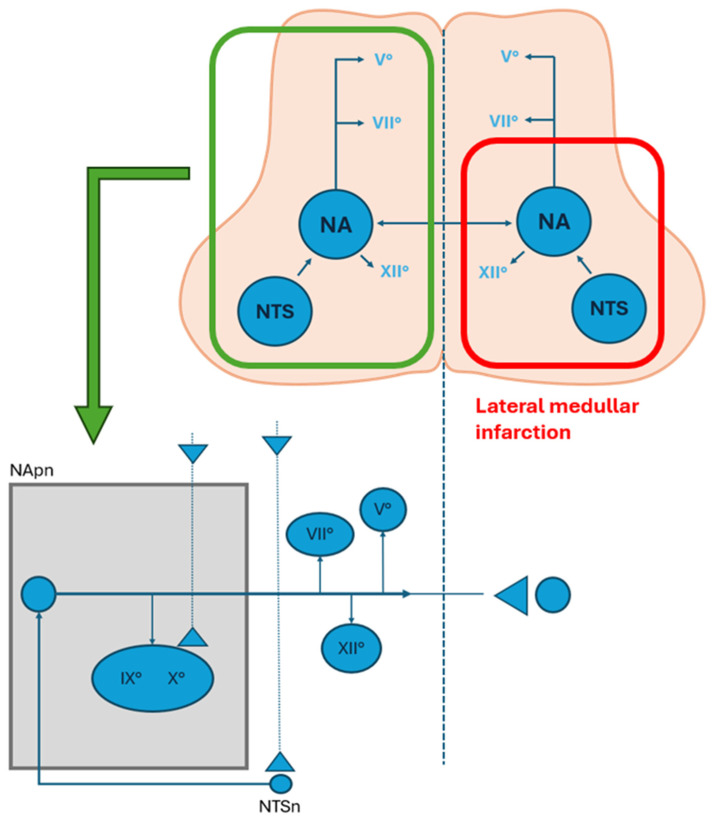
Representation of the swallowing centers and their connection with cranial nerves nuclei in the medulla oblonga. The area limited by the red line represents a lateral medullary infarction and the involvement of the nucleus of tractus solitarius (NTS) and the nucleus ambiguous (NA). The bottom figure represents the premotor neurons and their ipsilateral connection to the cranial nerve pools and the controlateral swallowing center (modified from Aydogdu et al., 2001) [31]. (NA: nucleus ambiguus; NTS: nucleus of tractus solitarius; NTpn: nucleus ambiguus premotor neurons; NTSn: nucleus tractus solitarius neurons).

In early stage of LMI, dysphagia may result from regional disconnection between the two hemi-CPG swallowing networks, leading to severe pharyngeal dysfunction. Notably, dysphagia in LMI is more frequent in rostral lesions, and some studies suggest compensatory cortical plasticity (Figure 2).

LMI also tends to produce dysphagia more often than medial medullary infarction (MMI). The onset of dysphagia following pontine infarction, which usually is unilateral, suggests a role for the pontobulbar trigeminal system in sustaining swallowing function. Sensory deficits in afferent pathways—originating from oropharyngeal structures and conveyed via trigeminal, glossopharyngeal, and vagal nerves—may contribute to dysphagia and aspiration in these patients.

The phenotypic profile of dysphagia following brainstem strokes, particularly when involving the medulla oblongata, is characterized by a delayed or absent swallowing reflex. These alterations occurred in over half of the patients studied by Warnecke et al. (2021) [25]. Additionally, residue in the piriform sinus, indicating dysfunction of the UES, is frequently reported. This pattern contrasts with the vallecular residue typically seen in Parkinson’s disease or ALS. It is likely due to impaired relaxation of the CP muscle, which can be attributed to disrupted transmission from the CPG to the cranial nerve nuclei responsible for coordinating the pharyngo-esophageal segment. FEES findings in brainstem strokes may also reveal CP spasm, impaired secretion management, and, in some cases, pharyngolaryngeal movement disorders if extrapyramidal structures are affected.

The pathophysiology underlying dysphagia in brainstem stroke often involves both motor and sensory components. Pharyngeal hypoesthesia, due to sensory deficits following the stroke, can prevent the initiation of the swallowing reflex even when the bolus has reached the appropriate trigger zone in the pharynx. This sensory impairment contributes to post swallow aspiration, as bolus material remains unrecognized and uncoordinated with airways protection mechanisms. Particularly in LMI, interruption of afferent and efferent pathways essential to the medullary swallowing CPG activity leads to severe and persistent dysphagia. In many cases, recovery is incomplete, and early rehabilitation, compensatory strategies, and artificial feeding are necessary.

Clinically, these distinctions carry significant implications. In ALS, the progressive and multifocal degeneration results in a heterogeneous and evolving dysphagia pattern, making ongoing assessment crucial. Swallowing impairment may necessitate early PEG placement and close monitoring of respiratory–swallowing interaction. Conversely, in brainstem stroke, dysphagia severity and recovery trajectory are often determined by lesion location and extent of brainstem involvement. While some patients experience improvement over time, others, especially those with bilateral or medullary lesions, may face long-term dependence on enteral feeding.

In summary, while both ALS and brainstem stroke can cause severe dysphagia, they differ in onset, mechanism, and clinical course. ALS leads to a progressive, multifactorial impairment that typically involves both oral and pharyngeal phases, often complicated by CPG dysfunction and hyperreflexic CP muscle. Instead, brainstem stroke, especially when involving the medulla, more commonly results in reflex delays, pharyngeal hypoesthesia and UES dysfunction. Recognizing these disease-specific dysphagia phenotypes enhances diagnostic precision and allows for more tailored, pathophysiologically grounded interventions (Table 2).

## 5. Therapies

A better understanding of brainstem networks may enable more targeted therapies for conditions such as Parkinson’s disease, ALS, multiple sclerosis, and, notably, brainstem stroke. As previously discussed, the brainstem contains a swallowing control center that includes the NA, the NTS, and the surrounding reticular formation. Consequently, a stroke affecting the medulla oblongata is referred to as “true bulbar paralysis” [30].

The severity and prognosis of post-stroke dysphagia largely depend on lesion size and precise location, as shown in brainstem MRI. However, MRI reveals only the infarcted region, not the specific structures involved in swallowing, limiting its utility in explaining the variability in dysphagia severity among patients with LMI [32]. In the future advanced techniques (e.g., fMRI of brainstem networks, DTI for axonal integrity) might improve diagnosis of brainstem damage.

Currently, there are no standardized treatment protocols for dysphagia caused by brainstem lesions. Most evidence referring to stroke is anecdotal and based on individual clinical cases using varied therapeutic approaches.

Dysphagia management generally includes conventional therapies such as dietary adjustments, exercises to strengthen oropharyngeal muscles and enhance expectoration, compensatory strategies to support laryngeal motion, and techniques to trigger the swallowing reflex. More advanced approaches involve neuromuscular electrical stimulation targeting the suprahyoid and/or infrahyoid muscles to promote anterior hyoid movement. A recent review reported that in post-stroke dysphagia neuromuscular electrical stimulation coupled with traditional swallowing therapy could be an optional intervention to improve swallowing function [33].

Beyond general approaches, several specific modalities have been explored. Non-invasive treatments include repetitive transcranial magnetic stimulation (rTMS) and transcranial direct current stimulation (tDCS), both of which show increasing evidence of neural remodeling that may improve motor, cognitive, and speech functions [34,35]. Recent applications of rTMS and tDCS in stroke-related dysphagia, including in LMI, have shown that these methods can enhance cortical excitability and modulate swallowing-related neural networks [36,37,38,39]. However, the effectiveness of neurostimulation for poststroke dysphagia is not well-documented by evidence-based medicine. Particularly, there is a need for more standardized, large-scale, and well-designed randomized controlled trials, especially for brainstem stroke patients, as current research often focuses on cortical or subcortical strokes [40,41]. Moreover, while some non-invasive brain stimulation techniques show promise, high heterogeneity in protocols and a lack of focus on specific brainstem lesions limit strong conclusions and clinical application for brainstem stroke dysphagia [42].

Rehabilitation strategies such as the Mendelsohn maneuver, lingual exercises, and Shaker exercises complement these interventions, targeting pharyngeal weakness or impaired UES function. A novel maneuver known as “vacuum swallowing” which induces negative esophageal pressure via diaphragmatic contraction before swallowing, has shown promise [43]. There is also increasing discussion about the potential benefits of lingual resistance training in patients with ALS. Although the application of exercise-based interventions has traditionally been controversial in this population, recent perspectives suggest that low-load exercise programs may be safe and beneficial, including for bulbar functions [44]. Preliminary studies in other populations have demonstrated that lingual resistance training can improve tongue strength, hyolaryngeal elevation, UES opening, and reduce pharyngeal residue. However, in ALS, its effectiveness remains to be confirmed.

On the other hand, invasive treatments involve interventions such as neurobotulinum toxin-A (BoNT-A) injections, balloon catheter dilation, and CP myotomy to facilitate UES opening and reduce its resistance [29]. Although these treatments have shown efficacy in diverse neurological conditions [45,46,47,48,49], specific data on LMI related dysphagia is limited and there is no evidence of their effectiveness [29]. Regarding BTX-A, few studies have reported on its effect in patients with LMI. Among these studies, small sample sizes prevail. In addition, injection schemes were variable, the effects of BTX-A weak, and the outcome definition different [29]. Balloon dilation has also shown potential in isolated LMI reports [50,51], but controlled studies are lacking. Finally, CP myotomy has benefited patients unresponsive to other methods [52,53], yet carries risks such as esophagopharyngeal reflux and nerve injury [54]. Nevertheless, given the possibility of spontaneous recovery in some cases up to three months post-stroke, more robust evidence is needed to clarify indications and risks for each treatment.

Finally, since stroke represents a long-term condition with recovery times that are not always predictable, structured and prolonged follow-up is of utmost importance. Particularly, the long-term follow-up represents a fundamental component of post-stroke care, as it allows for the timely identification and management of complications, supports the optimization of secondary prevention, and addresses evolving rehabilitation needs. A multidisciplinary approach, involving coordinated efforts of neurologists, nutritionists, speech therapists, nurses, and other healthcare professionals, is essential to ensure comprehensive and patient-centered care. Such integrated programs not only strengthen clinical outcomes but also may reduce the overall long-term health burden of stroke.

## Figures and Tables

**Table 1:** Causes (and brief characteristics) of brainstem damage.

**VASCULAR—BRAINSTEM STROKE**	**Hemorragic stroke**	Lesions of basilar artery or posterior cerebral arteries (PCA) or vertebral arteries or posterior inferior cerebellar artery (PICA) or anterior inferior cerebellar artery (AICA) or small penetrating arteries
	**Ischemic stroke (Wallenberg’s Syndrome)**	Dissection of the vertebral artery or atherothrombotic occlusion of posterior inferior cerebellar artery (PICA)
**DEMYELINATING**	**Multiple Sclerosis (MS)**	Chronic inflammation from MS damages the myelin sheathes that cover nerves, causing lesions
**DEGENERATIVE**	**Kennedy’s disease (or Spinal and Bulbar Muscular Atrophy)**	Is a rare, X-linked disorder characterized by degenerative process of anterior horn cell and dorsal root ganglion without upper motor neuron dysfunction
	**Amyotrophic Lateral Sclerosis (ALS)**	Brainstem lesions characterized by neuronal loss and gliosis
	**Parkinson’s disease**	Lewy bodies, abnormal protein deposits in brainstem areas
	**Progressive Bulbar Palsy**	Is a variant form of ALS, characterized by degeneration of lower motor neurons of brainstem
**MALFORMATION**	**Arnold-Chiari Syndrome**	Compression of brainstem
**TOXIC**	**Opioids, sedatives/hipnotics, stimulants, psychedelics**	Hypoxic injury for overdose
**INJURIES**	**Traumas**	Iatrogenic (posterior fossa and skull base surgery)
**INFECTIONS**	**Encephalitis or Meningitis**	
**EXPANSIVE PROCESSES**	**Brainstem Tumors**	

**Table 2:** Amyotrophic lateral sclerosis (ALS) vs. Brainstem Stroke FEES findings.

	**AMYOTROPHIC LATERAL SCLEROSIS** **(ALS)**	**BRAINSTEM STROKE**
**MAIN DYSPHAGIA PHNEOTYPE**	Complex swallowing disorder (48% of cases)	Delayed swallowing reflex (53% in infratentorial stroke) and residue in the piriform sinus
**COMMON FEES FINDINGS**	- Pharyngeal residue (valleculae & piriform sinus) - Premature bolus spillage - Reduced pharyngeal contractility - Always with oral phase involvement	- CP spasm - Pharyngolaryngeal movement disorders - Post-deglutitive aspiration - UES dysfunction
**CPG (SWALLOWING CENTRAL PATTERN)**	- Possibly impaired due to loss of corticobulbar control and interneuron degeneration	- Impaire due to direct lesion affecting the CPG or its connections to cranial nerve nuclei
**UNIQUE ELECTROPHYSIOOLOGICAL PBSERVATIONS**	- Irregular Sequential Water Swallowing (SWS) patterns in 43% - Hyperreflexic CP-MEG (premature closure, unexpected bursts)	- Subclinical or transient dysphagia possible - Severity varies with lesion location (e.g., medulla oblongata = more severe)
**PROGNOSIS FOR SWALLOWING FUNCTION**	Progressive decline; PEG often needed in advances stages	Variable; Lateral medullary infarction may have better prognosis than medial strokes
**THERAPEUTIC CONSIDERATIONS**	Focus on compensatory strategies, PEG, EMG monitoring	Early detection via electrophysiology, FEES, targeted swallowing therapy

## Data Availability

The original contributions presented in this study are included in the article. Further inquiries can be directed to the corresponding author(s).

## Abbreviations

The following abbreviations are used in this manuscript:

ALS	amyotrophic lateral sclerosis
BoNT-A	neurobotulinum toxin A
CP	cricopharyngeal
CP-EMG	cricopharyngeal electromyography
CPG	central pattern generator
DSG	dorsal swallowing group
EEG	electroencephalogram
EMG	electromyography
FEES	flexible endoscopic evaluation of swallowing
GPC	central pattern generator
LMI	lateral medullary infarction
MMI	medial medullary infarction
NA	nucleus ambiguus
ND	neurogenic dysphagia
NTS	nucleus tractus solitarius
PEG	percutaneous endoscopic gastrostomy
REM	rapid eye movement
rTMS	repetitive transcranial magnetic stimulation
SM-EMG	submental/suprahyoid electromyography
SS	spontaneous swallowing
tDCS	transcranial direct current stimulation
UES	upper esophageal sphincter
VSG	ventral swallowing group
